# Analysis of different adipose depot gene expression in cachectic patients with gastric cancer

**DOI:** 10.1186/s12986-022-00708-x

**Published:** 2022-10-31

**Authors:** Jun Han, Zuoyou Ding, Qiulin Zhuang, Lei Shen, Fan Yang, Szechun Sah, Guohao Wu

**Affiliations:** 1 Department of General Surgery, Zhongshan Hospital, Fudan University, 180 Fenglin Road, Shanghai, 200032 China; 2 Shanghai Clinical Nutrition Research Center, Shanghai, China

**Keywords:** Cancer cachexia, Adipose tissue loss, Subcutaneous adipose tissue, Visceral adipose tissue, Iroquois homeobox 1

## Abstract

**Purpose:**

This study aimed to identify the differentially expressed genes (DEGs) that contributed to the different amount of fat loss between subcutaneous adipose tissue (SAT) and visceral adipose tissue (VAT) among cachectic patients.

**Methods:**

RNA sequencing was performed and bioinformatic tools were utilized to analyze the biological functions and construct regulation networks of DEGs. We presumed that iroquois homeobox 1 (IRX1) to be a hub gene and analyzed its clinical significance. Mouse model of cancer cachexia was established and differences between SAT and VAT were compared. The function of IRX1 on lipid metabolism was clarified by Oil Red O staining, qRT-PCR, and Western blotting in adipocytes.

**Results:**

A total of 455 DEGs were screened between SAT and VAT in cachectic patients. Several hub genes were selected and IRX1 was presumed to contribute to the pathological difference between SAT and VAT in cancer cachexia. Patients with higher expression of IRX1 in SAT than VAT revealed significantly higher weight loss, IL-6 and TNF-α, as well as lower BMI, SAT, and VAT area. IRX1 expression in SAT was negatively correlated with SAT area. In cachectic mice, the expression of IRX1 in SAT was significantly higher than that in VAT. The inhibition effect on adipogenesis exerted by IRX1 was also proved in vitro.

**Conclusion:**

These data supported that DEGs contribute to the different degrees of fat loss among adipose depots in cachectic patients. IRX1 in SAT promoted fat loss by inhibiting adipocyte differentiation and adipogenesis.

**Supplementary Information:**

The online version contains supplementary material available at 10.1186/s12986-022-00708-x.

## Introduction

Cancer cachexia is a clinical syndrome characterized by muscle atrophy and fat loss [1]. In cachectic patients, fat loss is a significant prognostic factor as it reduces chemotherapy tolerance [2]. Our previous studies have also shown that fat loss is related to poor nutritional status and high inflammatory factors in patients with cancer cachexia [3, 4]. It is also reported that fat loss occurs before the appearance of muscle atrophy in cachectic patients [5]. It is essential to understand the function and mechanism of fat loss in cancer cachexia, which represents a meaningful feature.

The anatomical location of the adipose depot influences lipid metabolism and function. SAT comprises more than 80% of the total body fat, whereas VAT comprises up to 10% and 20% of the total body fat, respectively [6]. Computed tomography (CT) image analysis enables precise quantification of adipose depots and has emerged as the golden standard to segment SAT and VAT [7]. Not only do SAT and VAT differ in anatomic location, but also in endocrine function, adipokine secretion and lipolytic activity [8, 9]. For example, the accumulation of VAT contributes to obesity related diseases, such as metabolic syndrome [10]. Accumulation of VAT is also a high-risk factor for colorectal cancer and other tumors [11, 12]. On the contrary, SAT accumulation is unlikely to exert negative effect on metabolism of patients. These metabolic differences demonstrate the necessity for evaluation based on adipose depots rather than total adipose tissue. Our previous research has already found that the loss of SAT was greater than the loss of VAT in a cohort study of 411 gastric cancer patients with cachexia [13]. And the SAT area index was an independent risk factor for poor prognosis in patients with gastric cancer cachexia. Hence, it is rather essential to evaluate the DEGs between SAT and VAT, and clarify the hub genes that promote the greater loss of SAT than VAT. Although genes were reported to be differentially expressed in SAT and VAT in obese patients [14], the DEGs between SAT and VAT in gastric cancer patients with cachexia has not been fully explored before.

It has been confirmed that the main mechanisms of adipose tissue loss in cancer cachexia include activation of lipolysis, white-to-brown trans-differentiation of WAT (WAT browning), and dysfunctional adipogenesis [15]. Our previous research has proved these points from multiple perspectives. Interleukin-6 (IL-6) was proved to induce adipose tissue loss in patients with cancer cachexia by promoting lipolysis and WAT browning [4]. LncRNA-CAAlnc1 could suppress adipogenesis by blocking the binding of HuR in adipose tissue of cancer cachexia patients [16]. MiR-410-3p could down-regulate IRS-1 and PPAR-γ to inhibit adipogenesis and lipid accumulation [17]. CircPTK2 was upregulated in adipose tissues of cachectic patients and induced adipose tissue loss by promoting lipolysis and inhibiting adipogenesis [18]. However, these studies have mainly focused on the DEGs of SAT between cachectic patients and non-cachectic patients. The DEGs between SAT and VAT in cachectic patients and the hub genes among them remain unclear.

In this study, RNA sequencing was performed to screen DEGs between SAT and VAT of gastric cancer patients with cachexia. Clinicopathological significance of DEGs was analyzed in a large number of samples of gastric cancer patients with cachexia. In addition, DEGs were further verified in cancer cachexia mouse models. The function of the DEGs was analyzed by gain and loss of function in preadipocytes.

## Materials and methods

### Patients

Gastric cancer patients with cachexia who underwent surgery in Zhongshan Hospital of Fudan University from June 2019 to December 2020 were included in this study. Inclusive criteria for the study were: (1) patients diagnosed with gastric adenocarcinoma cancer (not gastric stromal tumor or lymphoma); (2) patients received surgical treatment without preoperative radiotherapy or chemotherapy; (3) patients with abdominal CT examinations and complete clinical data; (4) patients with weight loss > 5% in recent 6 months before surgery. This study was approved by the Ethics Committee of Zhongshan Hospital of Fudan University (B2019-193R). Written informed consents were obtained from all patients.

### Clinical data collection

Height, weight, gender, and age were extracted from the preoperative medical records. Body mass index (BMI) was calculated as body weight (kg)/height^2^ (m^2^). Cachexia-related indicators were extracted from preoperative blood biochemical examinations. Inflammatory markers were measured with ELISA kit by expert from clinical laboratory. Areas of SAT and VAT from CT scans at the third lumbar vertebra were measured as described before [13].

### Human tissue specimens

Adipose tissues were obtained from enrolled patients. At the beginning of the operation, about 500 mg of SAT near the median abdominal incision was obtained. About 500 mg of omental adipose tissue was taken as VAT within 30 min after gastric cancer specimens were isolated. The adipose tissue was immediately stored in liquid nitrogen at − 80 °C or transferred into tissue fixative for further analysis.

### Mouse model of cancer cachexia

The mouse model of cancer cachexia refers to the previous methods [4]. In brief, Cachectic mice were induced by subcutaneous injection of colon-26 adenocarcinoma cells into the right flank of the mice. The littermate control mice received PBS injection only. Mice were euthanized at day 21 post-injection and were dissected to harvest inguinal white adipose tissue (iWAT) and epididymal white adipose tissue (eWAT). Weight of iWAT and eWAT was recorded at day 0 (control mice) and day 21 (cachectic mice). The proportion of adipose tissues loss was calculated by weight change (weight at day 0 subtract weight at day 21) divided to initial weight. All animal studies were performed in accordance with the guidelines provided by Animal Care Committee of Fudan University.

### RNA sequencing

The total RNA was extracted from 3 paired SAT and VAT from gastric cancer patients with cachexia. After quantification and qualification, a total amount of 1 µg RNA per sample was used as input material for the RNA sample preparations. Sequencing libraries were generated using NEBNext UltraTM RNA Library Prep Kit for Illumina (NEB, USA) following manufacturer’s recommendations and index codes were added to attribute sequences to each sample. The clustering of the index-coded samples was performed on a cBot Cluster Generation System using TruSeq PE Cluster Kit v3-cBot-HS (Illumina, USA) according to the manufacturer’s instructions. After cluster generation, the library preparations were sequenced on an Illumina Novaseq platform and 150 bp paired-end reads were generated. FeatureCounts v1.5.0-p3 was used to count the reads numbers mapped to each gene. Fragments per kilobase million (FPKM) of each gene was calculated based on the length of the gene and reads count mapped to this gene. The raw sequencing dataset that supported the results of this study was deposited in the NCBI GEO database. The data are accessible through GEO: GSE186466.

### RNA sequencing data analysis

Differential expression analysis of two groups was performed using the DESeq2 R package. Differentially expressed transcripts between the two groups were identified when |log Fold Change| > 0 and the *p* value < 0.05. Gene Ontology (GO) enrichment analysis of DEGs was implemented by the ClusterProfiler R package, in which gene length bias was corrected. We also used ClusterProfiler R package to test the statistical enrichment of differential expression genes in KEGG pathways. GO terms and KEGG pathways with corrected *p* value < 0.05 were considered significantly enriched by differential expressed genes.

Conventional enrichment analysis based on hypergeometric distribution depends on significantly up-regulated or down-regulated genes, and it is easy to omit some genes with insignificant differential expression but important biological significance. Gene set enrichment analysis (GSEA) does not need to specify a clear differential gene threshold. All genes are sorted according to the degree of differential expression in the two groups of samples, and then statistical methods are used to test whether the preset gene set is enriched at the top or low section of the sorting table. GSEA mainly includes three steps: calculation of enrichment score; estimation of the significance level of enrichment score; multiple hypothesis tests.

The PPI network of DEGs was predicted using the Search Tool for the Retrieval of Interacting Genes (STRING) database. The interaction score threshold of 0.4 was set as the cut-off criterion. The PPI network was constructed using Cytoscape. Comprehensive experimentally validated miRNA-gene interaction data were collected from TargetBase. Transcription factor and gene target data derived from the ENCODE ChIP-seq data. Only peak intensity signal < 500 and the predicted regulatory potential score < 1 is used (using BETA Minus algorithm).

### Cell culture and differentiation

The mouse immortalized white preadipocytes were kindly provided by Professor Qiurong Ding from the Shanghai Institute of Nutrition and Health, Chinese Academy of Sciences. This cell line has been described previously and used in several studies to assess the effects of different factors on adipose differentiation and function [19]. The culture and differentiation methods of preadipocytes cell lines were as previously reported [18]. Mouse preadipocyte was cultured in high-glucose Dulbecco’s modified Eagle medium (DMEM) supplemented with 10% fetal bovine serum (FBS), 100 U/ml penicillin and 100 mg/ml streptomycin. All cells were kept in an atmosphere of 5% CO_2_ and 95% oxygen at 37 °C. Differentiation of preadipocytes was initiated by an induction medium (0.5 mM isobutyl-1-methylxanthine (IBMX), 5 mM Dexamethasone, 1 μm Rosiglitazone, 5 ug/ml Insulin), and replaced with a maintenance medium (5ug/ml Insulin) after 2 days for further differentiation.

### Plasmid and siRNA construction

The iroquois homeobox 1 (IRX1) expression plasmid pcDNA3.1þ/IRX1 and empty plasmid pcDNA3.1þ were designed and synthesized by GenePharma (Shanghai, China). Small interfering RNAs (siRNAs) targeting IRX1 were also designed by GenePharma. Cell transfection was conducted using Lipofectamine RNAiMAX Transfection Reagent kit (Invitrogen, USA) or Lipofectamine 2000 Transfection Reagent (Invitrogen, USA) according to the manufacturer’s instruction. Lipofectamine™ RNAiMAX transfection reagent was used to transfect 100 nM of siRNAs when cell density was approximately 70%.

### Hematoxylin-eosin and immunohistochemical staining

All the samples were transferred to tissue fixative after being harvested. The protocols were previously described [18]. In brief, histological sections of adipose tissue were stained with hematoxylin–eosin to evaluate morphological changes and the adipocyte cross-sectional area (CSA). A total of 10 randomly selected fields for each section were captured and analyzed to evaluate adipocyte CSA with a computerized imaging software (ImageJ, USA). For immunohistochemistry, the positive cells in 10 randomly selected fields per section were counted and evaluated by two independent researchers. And the mean number of positive cells per field was calculated.

### Oil Red O (ORO) staining

Mature adipocytes were fixed with 4% formaldehyde for 30 min, then washed twice with PBS. They were stained with 0.3% ORO solution and washed three times with distilled water. To assess lipid accumulation, the dye retained in the cells was dissolved in isopropanol and the absorbance of the resulting solution at 520 nm was examined.

### RNA isolation and qRT-PCR

Total RNA was isolated from adipose tissues and adipocytes using TRIzol Reagent (Invitrogen, USA) according to the manufacturer’s recommendations. cDNA was synthesized from 1 µg total RNA using FastKing RT Kit (Tiangen, China). Gene expression analysis was performed using Prime-Script RT master mix (Takara, Japan) in StepOnePlus Real-Time system (Applied Biosystems, USA). Expression levels of targeted genes were normalized to the expression of GAPDH. qRT-PCR was performed according to the manufacturer’s instructions and the relative fold change was calculated by the 2^−ΔΔCt^ method. Primers were designed and synthesized by Sangon Biotech (Shanghai, China) and are listed in Additional file 2: Table S2. All experiments were repeated three times.

### Western blot analysis

Preparation of total protein lysates and western blot analysis were performed as previously described [16]. Primary antibodies against IRX1 (Immunoway, YT2412), CEBPα (Cell signaling technology, #2295), Adiponectin (Cell signaling technology, #2789), and FABP4 (Cell signaling technology, #2120) were used. Tubulin expression was used as an endogenous control.

### Statistical analyses

Statistical analyses were performed using GraphPad Prism software. Data calculated from independent experiments were presented as the mean ± standard deviation and a student’s *t*-test was performed to compare the differences between two groups. To analyze the correlation between IRX1 mRNA levels in SAT and the clinicopathological factors in gastric cancer patients with cachexia, we divided 61 patients into two groups according to the IRX1 expression in SAT compared to VAT. Comparisons between these two groups were made using the *t*-test for continuous data and χ^2^ test for categorical data. *p <* 0.05 was considered statistically significant.

## Results

### Identification of DEGs between SAT and VAT

**Fig. 1:**
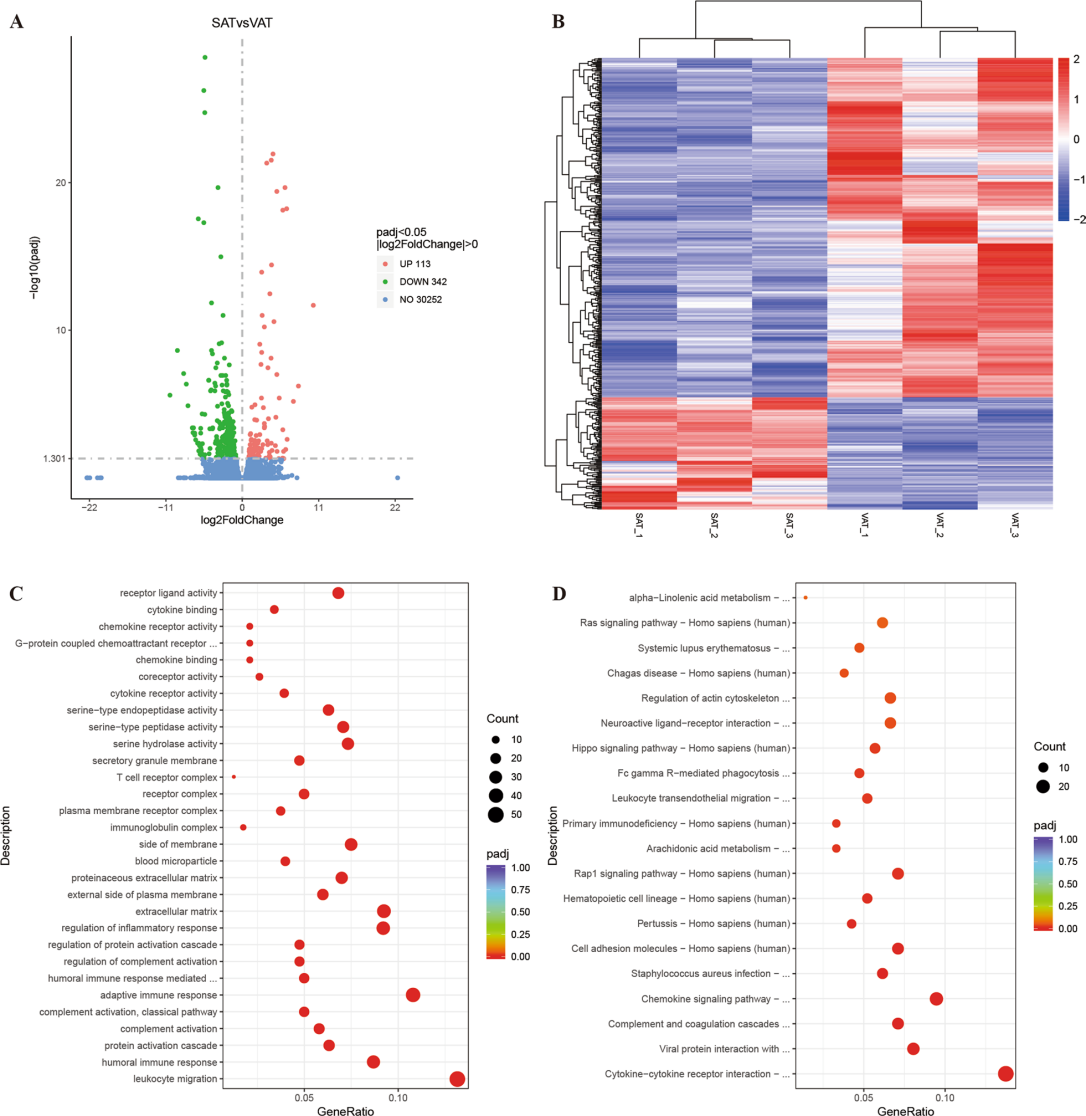
A total of 455 DEGs were identified as being statistically significant between SAT and VAT groups. **A** Volcano plot of differently expressed genes. **B** Heat map of differentially expressed genes. **C** Enriched GO terms. **D** KEGG pathway annotations. The X-axis represented the proportion of DEGs, and the Y-axis represented different categories

To investigate DEGs between SAT and VAT in patients with cachexia, we performed whole transcriptome RNA sequencing of SAT and VAT from three patients. Their clinical features and typical CT image at the third lumbar vertebra were shown in Additional File 2: Table S1 and Additional file 1: Fig. S1. Gene expression distributions of each sample were shown in Additional file 1: Fig. S2. As illustrated by volcano plot in Fig. 1 A, a total of 455 DEGs were detected in SAT compared to VAT (*p* < 0.05 and |log2FoldChange| > 0), including 342 downregulated mRNAs and 113 upregulated mRNAs. Cluster analysis of DEGs was shown in Fig. 1B by heatmap. The top 10 downregulated DEGs and top 10 upregulated DEGs were shown in Table 1. To better understand the functional implications of 455 DEGs between SAT and VAT in cachectic patients, we performed GO analysis, KEGG analysis, and GSEA analysis. The GO terms were shown in Fig. 1C. We found that the leukocyte migration, humoral immune response, complement activation, adaptive immune response, and humoral immune response mediated by circulating immunoglobulin were among the top 10 GO terms, which indicated that the immune response might play an essential role in different functions and mechanisms between SAT and VAT. Interestingly, cytokine-cytokine receptor interaction, complement and coagulation cascades, and chemokine signaling pathway were top 3 of the KEGG pathways (Fig. 1D), which also indicated the significance of immune response between SAT and VAT.

**Table 1:** Top 10 downregulated mRNAs and top 10 upregulated mRNAs in SAT compared to VAT

Gene id	Gene name	log2 fold change	*p* value
*Downregulated mRNAs*			
ENSG00000184937	WT1	− 10.42020286	7.24E−09
ENSG00000119919	NKX2-3	− 9.340609993	3.06E−12
ENSG00000183242	WT1-AS	− 8.433724032	1.59E−10
ENSG00000211892	IGHG4	− 8.083413442	1.00E−09
ENSG00000211668	IGLV2-11	− 7.842925465	4.15E−08
ENSG00000054803	CBLN4	− 7.209658278	2.30E−06
ENSG00000205038	PKHD1L1	− 7.107755456	5.64E−06
ENSG00000187848	P2RX2	− 7.045386839	2.30E−06
ENSG00000242221	PSG2	− 6.846739335	6.57E−06
ENSG00000211663	IGLV3-19	− 6.843669107	9.34E−06
*Upregulated mRNAs*			
ENSG00000112246	SIM1	10.22187292	1.84E−15
ENSG00000176842	IRX5	7.352859596	2.08E−08
ENSG00000089225	TBX5	6.447165153	2.06E−05
ENSG00000170549	IRX1	6.369599248	2.82E−22
ENSG00000180318	ALX1	6.250090981	0.000155648
ENSG00000180818	HOXC10	6.123438559	7.65E−24
ENSG00000251151	HOXC-AS3	5.976364812	0.001044655
ENSG00000154162	CDH12	5.92279196	0.00024887
ENSG00000255399	TBX5-AS1	5.831484757	3.40E−06
ENSG00000250451	HOXC-AS1	5.825341418	3.79E−22

### GSEA analysis and interaction network of DEGs

**Fig. 2:**
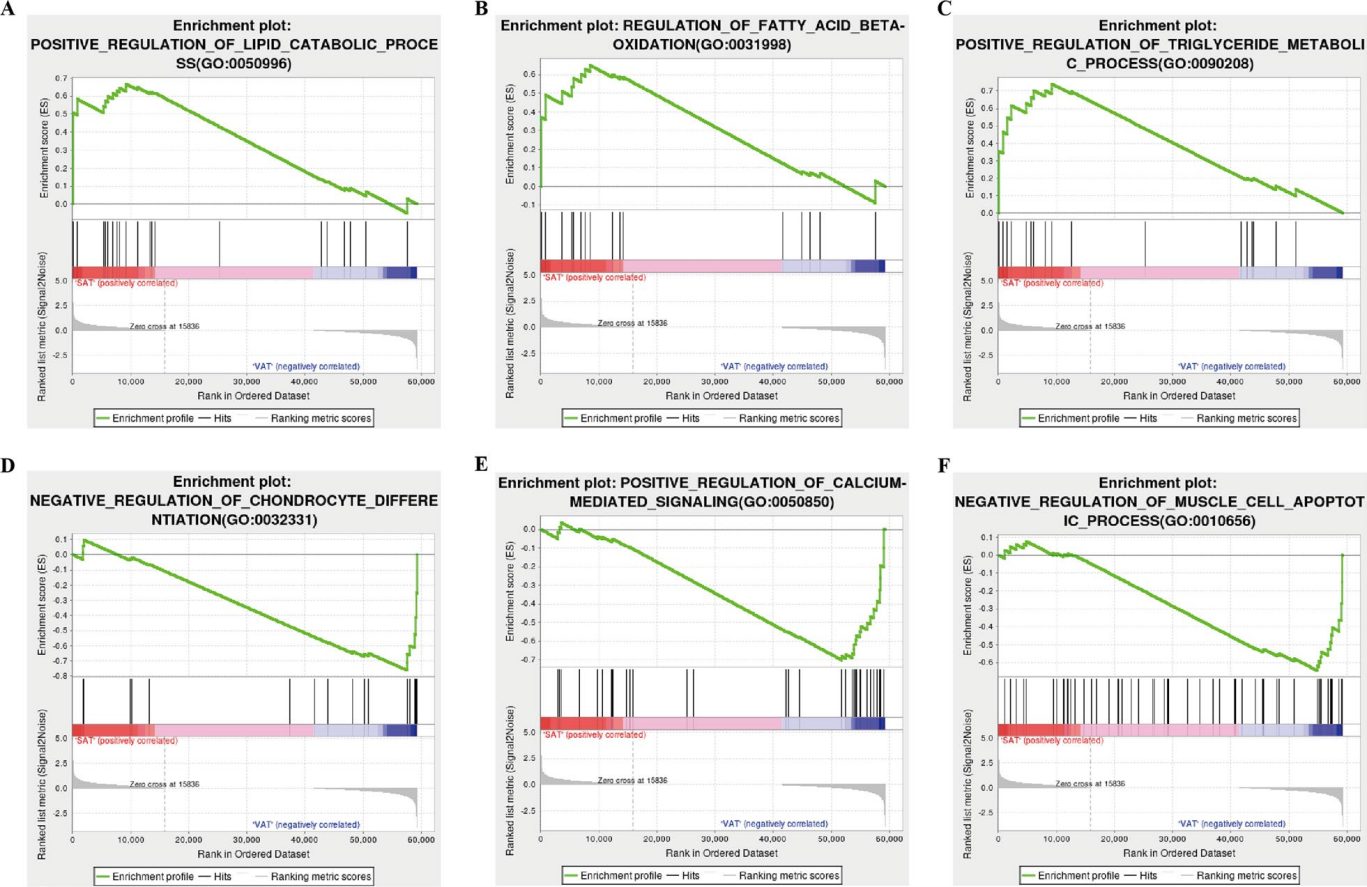
Enrichment plots from the gene set enrichment analysis (GSEA). **A–F** ES, enrichment score; NES, normalized ES; ADJ p-val, adjusted p-value

**Fig. 3:**
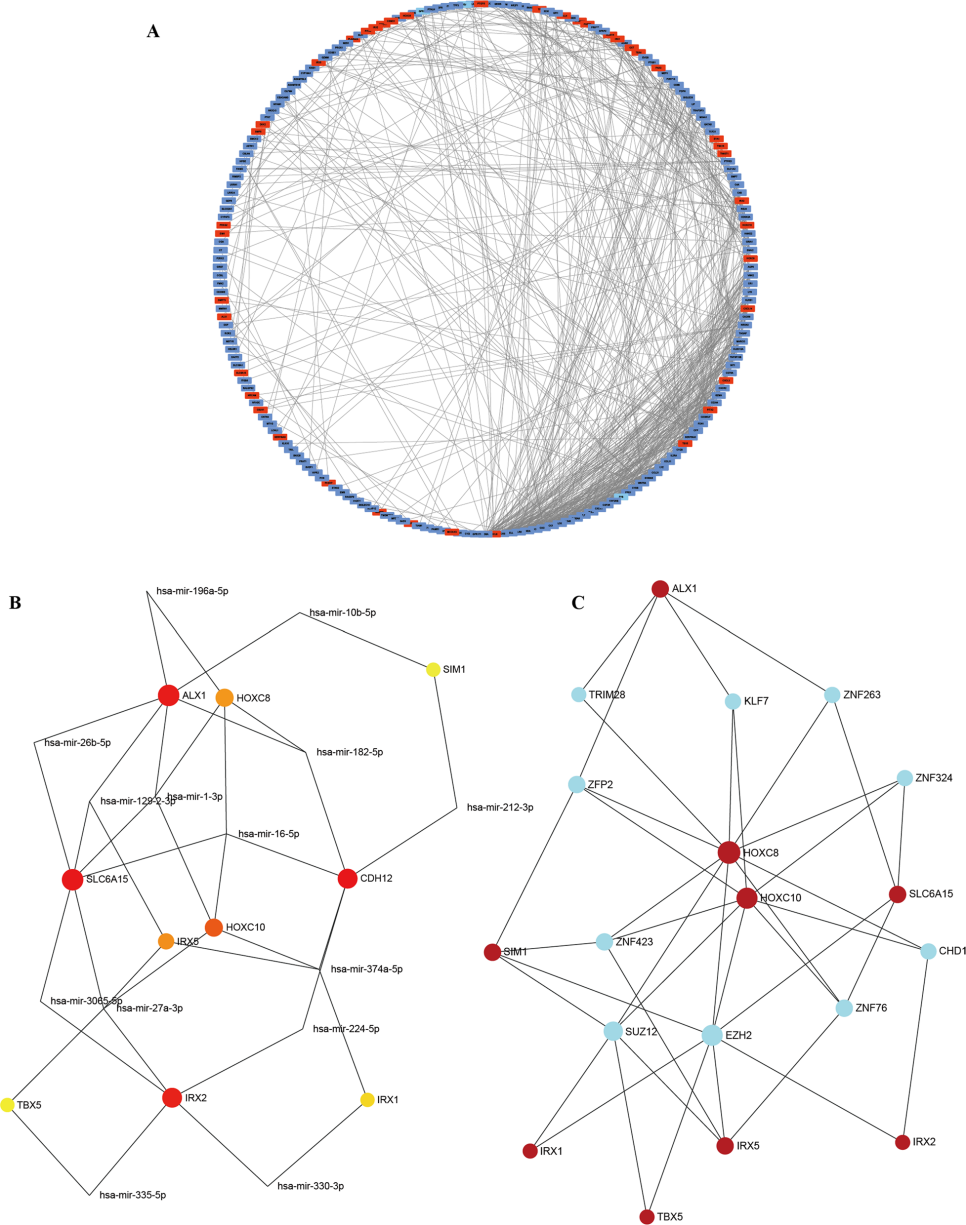
The PPI network and interaction network of DEGs between SAT and VAT. **A** The PPI network of DEGs was constructed using Cytoscape. **B** Interaction network linking top 10 DEGs to miRNAs. **C** Interaction network linking top 10 DEGs to translation factors

GSEA analysis was conducted to achieve further insight into the biologic pathways involved in different adipose depots of cachectic patients. As shown in Fig. 2 A–C, we found that lipid catabolic process, regulation of fatty acid beta-oxidation, and positive regulation of triglyceride metabolic process were enriched in SAT group. On the contrary, we found that negative regulation of chondrocyte differentiation, positive regulation of calcium-mediated signaling, and negative regulation of muscle cell apoptotic process were enriched in VAT group **(**Fig. 2D, F**)**. The network of DEGs between SAT and VAT was constructed by STRING, with a threshold of 0.4. The PPI network with 205 nodes and 720 edges was displayed by Cytoscape-MCODE (Fig. 3 A). Then we constructed an interaction network linking top 10 DEGs to their relevant miRNAs/translation factors. As shown in Fig. 3B, C, HOXC8, HOXC10, and IRX5 were the hub genes in the regulating network. IRX1, IRX2, TBX5, CDH12, SLC6A15, ALX1, and SIM1 also played key roles in this biological process.

### Expression of IRX1 was higher in SAT than VAT in cachectic patients

**Fig. 4:**
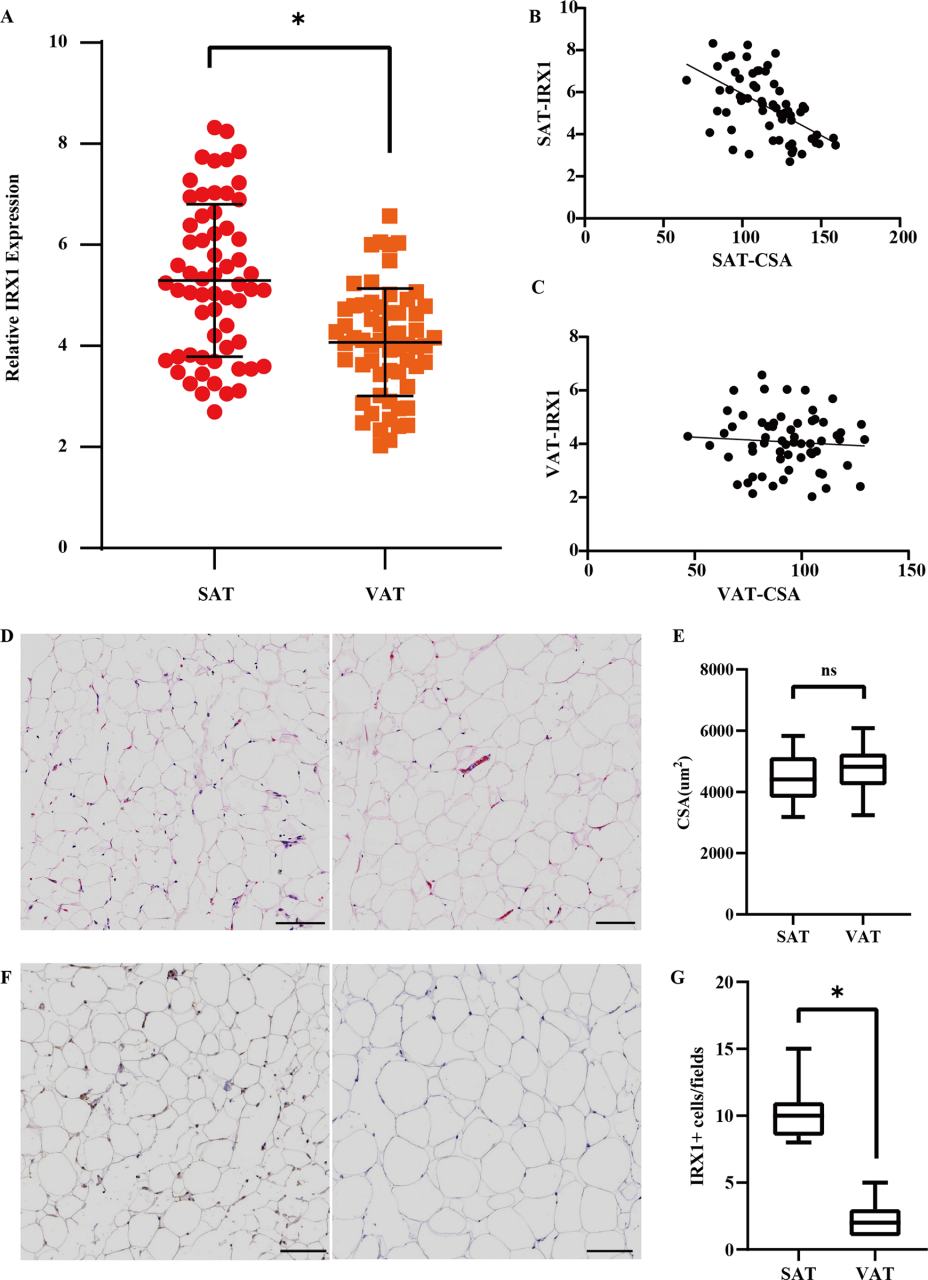
The expression of IRX1 was associated with pathological process in cancer cachexia. **A** Expression of IRX1 between SAT and VAT in 61 pairs of specimens. **B** Correlation between the SAT area and the expression level IRX1. **C** Correlation between the VAT area and the expression level IRX1. **D**, **E** Morphological evaluation of adipose tissue of SAT and VAT (hematoxylin–eosin) (Scale bar = 100 μm). **F**, **G** Representative images of IHC revealing IRX1 expression in SAT and VAT (Scale bar = 100 μm). * *p* < 0.05. ns indicates no significance

According to analysis above, we found that IRX1, not only highly expressed in SAT compared to VAT, but also revealed a potential in determining the different loss degree between SAT and VAT. To further clarify the clinical significance of different expression of IRX1 between SAT and VAT, we performed qPCR assay and showed that IRX1 was significantly upregulated in SAT compared with VAT (*p* < 0.05, Fig. 4A). Among the 61 pairs of adipose tissues, we found that IRX1 was higher expressed in SAT than VAT in 37 pairs and lower expressed in SAT than VAT in 24 pairs. Then, we divided them into two groups and analyzed the clinicopathological correlation between groups. The analysis showed that weight loss, IL-6, and TNF-α were significantly increased in cachectic patients with higher IRX1 expression in SAT than VAT. While BMI, SAT area and VAT area were significantly decreased in cachectic patients with higher IRX1 expression in SAT than VAT (Table 2). In addition, correlation analysis was performed between IRX1 expression and adipose tissue area. The results of the Spearman’s correlation analysis showed that the expression level of IRX1 in WAT was negatively correlated with WAT area (r = − 0.55, *p* < 0.001) (Fig. 4B), and the expression level of IRX1 in VAT revealed no correlation with VAT area (r = − 0.07, *p* = 0.58) (Fig. 4C). To further validate our hypothesis, HE staining and IHC were performed in WAT and VAT from cachectic patients. The representative images of HE staining revealed that there is no significant difference between adipocytes of SAT and VAT in terms of morphology (Fig. 4D). The representative images of IHC confirmed that there is a significant increase in IRX1 expression of adipocytes in SAT with respect to VAT (Fig. 4E, p < 0.05). These results suggested that IRX1 is involved in the lipid metabolism of the pathological process in cachexia.

**Table 2:** Clinical characteristics of 61 participants

Clinical characteristics	IRX1		*p* Value
	High (n = 37)	Low (n = 24)	
Age	64.71 ± 9.06	65.79 ± 8.38	0.633
BMI	20.36 ± 2.12	22.82 ± 1.61	0.006*
Weight loss	6.53 ± 2.19	5.12 ± 1.93	0.015*
Disease stage (III/IV)	23/37	14/24	0.772
IL6 (mmol/L)	10.67 ± 5.87	7.52 ± 4.38	0.027*
TNFa (mmol/L)	14.24 ± 6.36	11.80 ± 4.11	0.019*
Alb (g/L)	37.89 ± 3.43	39.04 ± 4.45	0.261
PAb (mg/L)	207.59 ± 52.98	220.17 ± 58.42	0.093
FAA (mmol/L)	0.58 ± 0.13	0.53 ± 0.17	0.098
TC (mmol/L)	4.01 ± 0.95	4.26 ± 1.05	0.351
TG (mmol/L)	1.07 ± 0.41	1.10 ± 0.37	0.829
LDL (mmol/L)	2.37 ± 0.84	2.60 ± 0.91	0.317
HDL (mmol/L)	1.18 ± 0.36	1.25 ± 0.57	0.599
ApoA (g/L)	1.16 ± 0.28	1.13 ± 0.26	0.445
ApoB (g/L)	0.79 ± 0.25	0.85 ± 0.21	0.268
ApoE (mg/L)	35.78 ± 14.30	39.06 ± 16.10	0.156
SAT area (cm^2^)	108.54 ± 19.18	127.09 ± 24.13	< 0.001*
VAT area (cm^2^)	87.83 ± 17.78	101.09 ± 19.85	0.015*

*BMI* Body mass index, *ALB* Albumin, *PAb* Prealbumin, *TC* Total cholesterol, *TG* Tri-glyceride, *LDL* Low-density lipoprotein, *HDL* High-density lipoprotein, *ApoA* Apolipoprotein A, *ApoB* Apolipoprotein B, *ApoE* Apolipoprotein E, *FFA* Free fatty acid, *IL-6* Interleukin 6, *TNF-α* Tumor necrosis factor-α

* *p* < 0.05

### Expression of IRX1 was higher in SAT than VAT in mouse model of cachexia

**Fig. 5:**
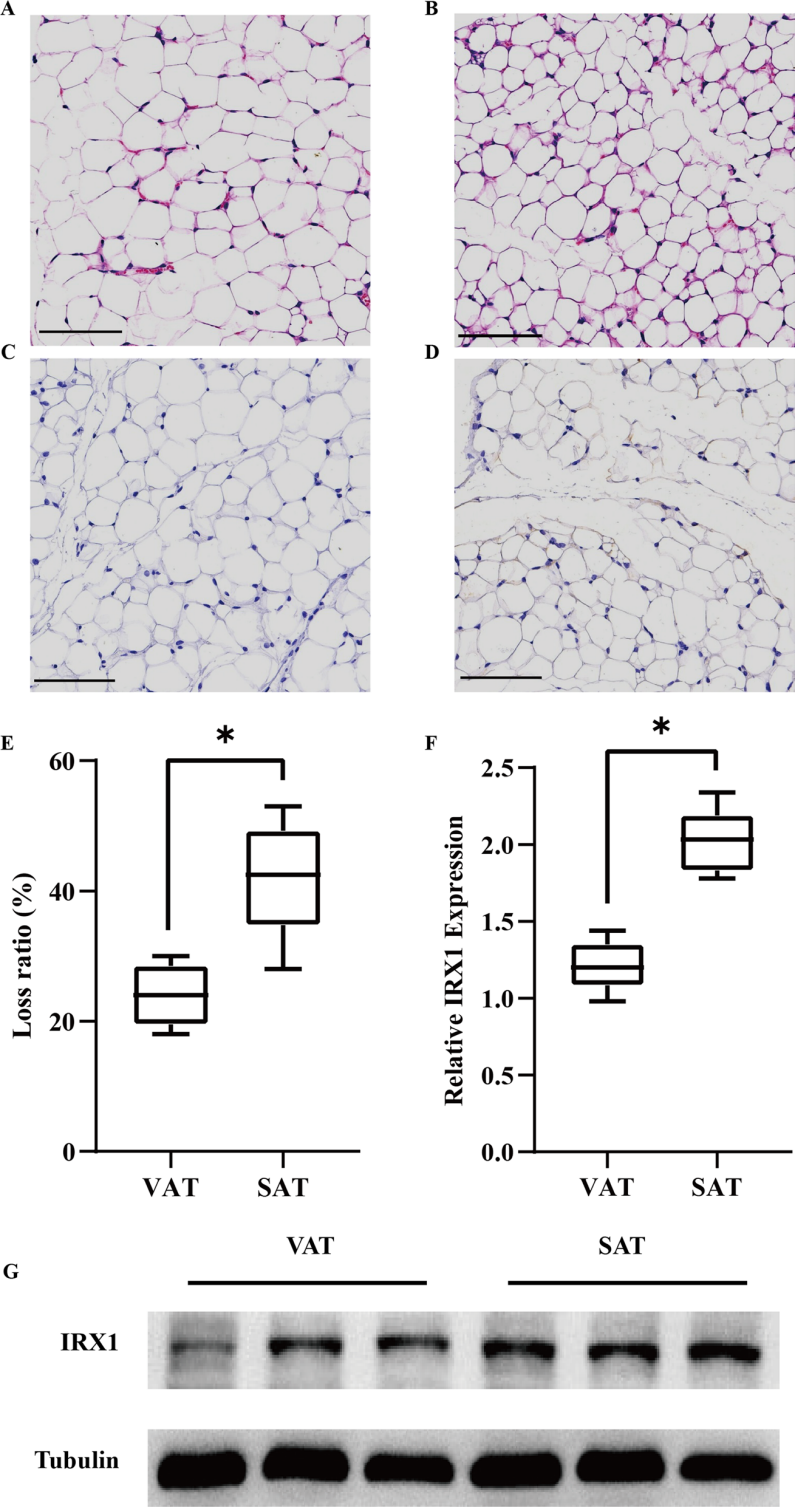
IRX1 was highly expressed in SAT of cachectic mice. **A**, **B** HE staining of SAT and VAT of mice (Scale bar = 100 μm). **C**, **D** Representative images of IHC revealing IRX1 expression in SAT and VAT (Scale bar = 100 μm). **E** Comparison of loss ratio of SAT and VAT in cachectic mice. **F** mRNA expression and **G** protein expression levels of IRX1 in SAT and VAT in cachectic mice. * *p* < 0.05

To further validate our findings in animal models, we constructed a mouse model of cancer cachexia by C26 cell injection. At 3 weeks post-injection, inguinal and epididymal part of mice were exposed. It could be observed that cancer cachexia mice revealed an obvious fat loss (Additional file 1: Fig. S3). As shown in Fig. 5A, B, it became clear that adipocytes morphology did not reveal significant difference between different fat depots of the same cachectic mice. Yet the IRX1 expression of adipocytes in SAT was significantly higher than that in VAT (Fig. 5C, D). To calculate the loss ratio of different fat depots, we record weight of SAT and VAT at day 0 (control mice) and day 21 (cachectic mice). The result showed that loss ratio of SAT was significantly higher than that of VAT in cachectic mice (Fig. 5E). Then we compared the mRNA and protein levels of IRX1 in SAT and VAT of cancer cachexia mice. The results showed that the mRNA and protein expression levels of IRX1 in SAT were higher than that in VAT in cancer cachexia mice (Fig. 5F, G), which was consistent with the results above.

### IRX1 inhibited adipogenesis and differentiation in adipocytes

**Fig. 6:**
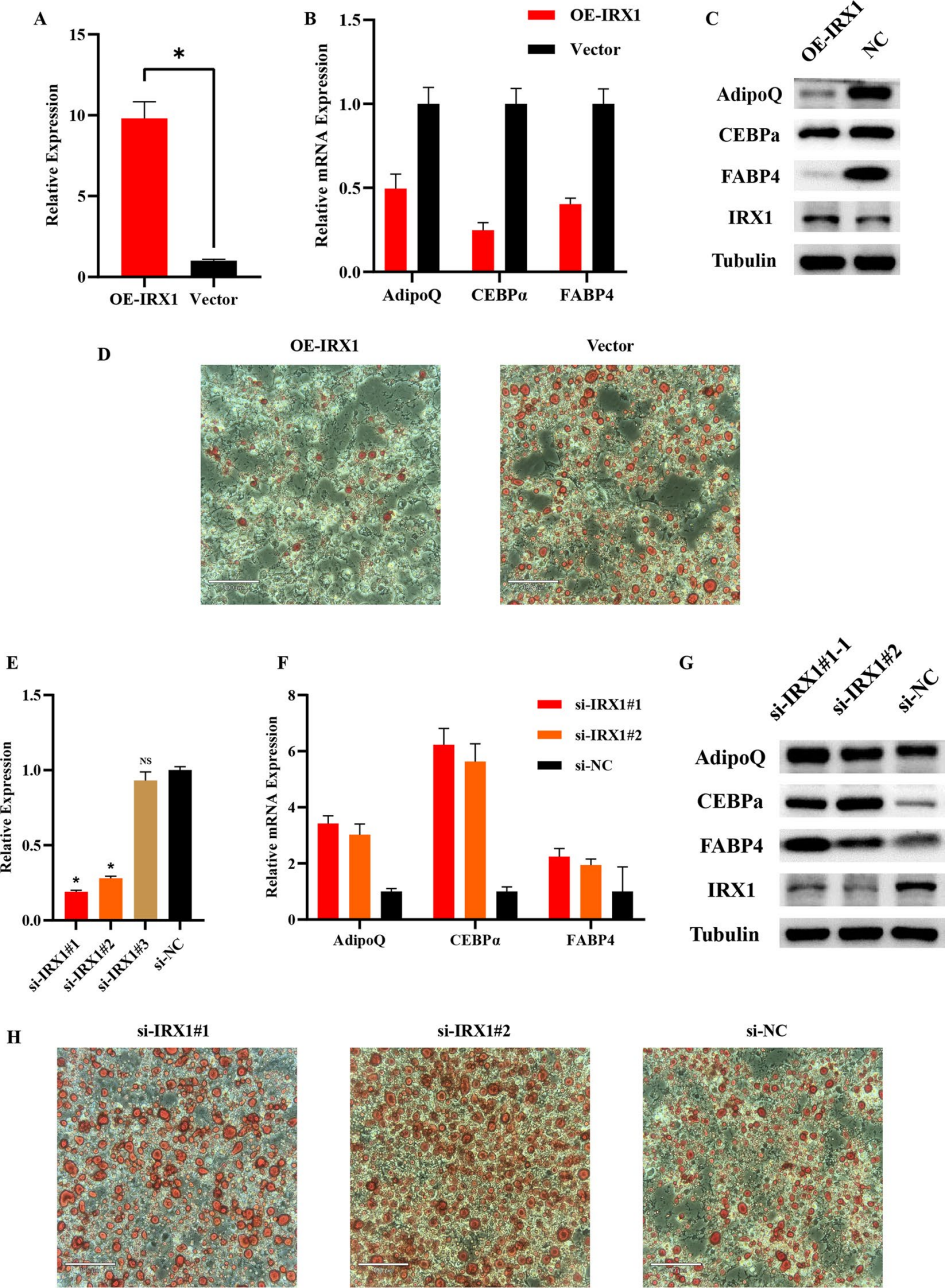
IRX1 suppresses adipogenesis in adipocytes. **A** Relative IRX1 expression with or without IRX1 overexpression. **B**, **C** qPCR and Western blot analysis of the expression of markers in adipocytes (6d) without/with overexpression of IRX1. **D** Oil red O staining of lipid accumulation in adipocytes (6d) without/with overexpression of IRX1 (Scale bar = 100 μm). **E** Relative IRX1 expression with or without IRX1 knockdown. **F**, **G** qPCR and Western blot analysis of the expression of markers in adipocytes (6d) without/with knockdown of IRX1. **H** Oil Red O staining of lipid accumulation in adipocytes (6d) without/with knockdown of IRX1 (Scale bar = 100 μm). * *p* < 0.05; ns indicates no significance

To investigate the role of IRX1 in adipocyte metabolism, IRX1 was upregulated by transfecting preadipocyte with IRX1 overexpression plasmid, followed by induction and differentiation. The qPCR results showed that IRX1 expression had increased about ten-fold by differentiation day 6 in the IRX1 overexpression group compared to the control group (Fig. 6A). We detected the changes of adipogenesis-related genes involved in the lipid metabolism after IRX1 overexpression. The results of qPCR and western blotting showed that Adiponectin, FABP4, and CEBPα were significantly down-regulated when IRX1 was overexpressed (Fig. 6B, C). IRX1 overexpression also resulted in decreasing in lipid droplet according to the ORO staining (Fig. 6D). To further demonstrate whether IRX1 was required for adipocyte metabolism, adipocytes were transfected with three independent siRNAs. Effective knockdown was achieved by two of the siRNAs (Fig. 6E). The results of qPCR and western blotting showed that Adiponectin, FABP4, and CEBPα were also significantly upregulated when IRX1 was knockdown (Fig. 6F, G). The results of ORO staining were consistent with the findings above (Fig. 6H).

## Discussion

The role of adipose tissue wasting in patients with cancer cachexia is becoming a heat topic. SAT and VAT play different roles in the development of cancer cachexia. However, there is no systematic study on DEGs between SAT and VAT in gastric cancer patients with cachexia. In this study, the gene expression profiles of SAT and VAT in patients with cancer cachexia were screened by RNA sequencing for the first time. 342 downregulated and 113 upregulated mRNAs were screened compared SAT to VAT in patients with gastric cancer cachexia. IRX1 was one of the most highly expressed mRNAs in SAT both in cachexic patients and mice. It is observed that weight loss, IL-6, and TNF-α were significantly increased while BMI, SAT area and VAT area were significantly decreased in cachectic patients with higher IRX1 expression in SAT than VAT. In vitro experiments showed that IRX1 promotes fat loss by inhibiting lipid differentiation and adipogenesis.

The metabolic differences between SAT and VAT were mostly studied in obese patients. The adipose tissues in both fat depots are similar in their morphology and lipid storage functions. As shown in present research, adipocytes from SAT an VAT in both cachectic patients and mice revealed evident atrophy, yet no significant difference was observed regarding the morphology of adipocytes. Nonetheless, emerging evidence has suggested that SAT and VAT have different characteristics and functions in lipid metabolism [20, 21]. The visceral depot is often considered more deleterious than the subcutaneous depot. Two independent research demonstrated that VAT hypertrophy is predominantly associated with dyslipidemia whereas SAT hypertrophy is mainly associated with insulin resistance in humans [22]. However, metabolic differences between SAT and VAT in cancer cachexia have not been reported. In this study, we detected hundreds of DEGs between SAT and VAT in gastric cancer patients with cachexia by mRNA sequencing. GO and KEGG analysis showed that immune response might be involved in the difference between SAT and VAT, which was consistent with the differential expression of immune cells in SAT and VAT by single cell sequencing [23]. However, the functions of specific types of immune cells and their mechanisms during the development of cancer cachexia remain further clarified.

IRX1, a member of the Iroquois homeobox family of transcription factors, played a crucial role in embryonic development [24]. Recently, IRX1 was identified as a potential tumor suppressor gene in head and neck squamous cell carcinoma and gastric cancer [25–27]. However, IRX1 also acted as a metastatic oncogene which was activated by hypomethylation [28]. As far as we know, the role of IRX1 in the progression of fat loss during cancer cachexia has not been elucidated. In this study, we found IRX1 was significantly upregulated in SAT compared with VAT in both human and mice. IRX1 expression in SAT was found to be negatively correlated with SAT area and was involved in the lipid metabolism of the pathological process in cachexia. These results indicated IRX1 gene plays an important role in promoting the loss of SAT during cancer cachexia.

We firstly reported that overexpression of IRX1 inhibited adipogenesis in preadipocytes. Activation of lipolysis and WAT browning or dysfunctional adipose differentiation and adipogenesis were reported to be the main mechanism of fat loss in cancer cachexia [29]. By overexpression and knockdown of IRX1, we clarified that IRX1 could inhibit adipogenesis in the lipid metabolism. These results suggested that adipose differentiation and adipogenesis related genes might be important in the regulation of lipid metabolism during cancer cachexia, which was consistent with our previous foundation. In future research, we will explore the specific molecular mechanism of IRX1 regulating adipose differentiation and adipogenesis related genes.

## Conclusion

In conclusion, we screened mRNA expression profiles of SAT and VAT in patients with cancer cachexia by RNA sequencing. Hundreds of DEGs were detected in SAT and VAT and IRX1 was one of the most differentially expressed mRNAs in SAT. Clinical studies showed that IRX1 expression in SAT was negatively correlated with SAT area. In vitro experiments showed that IRX1 inhibited lipogenesis by modulating differentiation and adipogenesis related genes. Our results demonstrated that IRX1 was expected to be a potential target for the prevention and treatment of SAT loss in cancer cachexia.

## Supplementary Information

**Additional File 1**. **Figure S1**: A representative CT image at the third lumbar vertebra (L3) marked with different body composition. Skeletal muscle (SM) in red; subcutaneous adipose tissue (SAT) in blue; visceral adipose tissue (VAT) in yellow; intramuscular adipose tissue (IMAT) in green. **Figure S2**: Gene expression distributions of each sample for RNA sequencing. **Figure S3**: Representative images of SAT and VAT from normal and cachectic mice.

**Additional File 2**. **Table S1**: Patients’ characteristics for RNA sequencing. **Table S2**: Primer Sequences used for qRT-PCR. **Table S3**: Clinical Characteristics of 61 cachectic patients.

## Data Availability

RNA sequencing data were deposited at the GEO database with the accession number GSE186466. All data generated or analyzed during this study are included in this published article and its supplementary information files.

## Abbreviations

WAT: White adipose tissue
SAT: Subcutaneous adipose tissue
VAT: Visceral adipose tissue
FPKM: Fragments per kilobase million
GO: Gene ontology
KEGG: Kyoto Encyclopedia and Genes and Genomes
GSEA: Gene set enrichment analysis
ORO: Oil red O
BMI: Body mass index
lncRNA: Long non-coding RNA
circRNA: Circular RNA
siRNA: Small interfering RNAs
ceRNA: Competing endogenouse RNA
IL-6: Interleukin-6
TNF-α: Tumor necrosis factor-α
FABP4: Fatty acid binding protein 4
CEBPα: CCAAT/enhancer binding proteinsα
IRX1: Iroquois homeobox 1
