# Supplementary material for: Analysis of different adipose depot gene expression in cachectic patients with gastric cancer
Source: Nutr Metab (Lond). 2022 Oct 31;19:72. doi: 10.1186/s12986-022-00708-x (PMC9624057; doi:10.1186/s12986-022-00708-x)
Supplement: Supplementary file 1 — Additional File 1. Figure S1: A representative CT image at the third lumbar vertebra (L3) marked with different body composition. Skeletal muscle (SM) in red; subcutaneous adipose tissue (SAT) in blue; visceral adipose tissue (VAT) in yellow; intramuscular adipose tissue (IMAT) in green. Figure S2: Gene expression distributions of each sample for RNA sequencing. Figure S3: Representative images of SAT and VAT from normal and cachectic mice. [file 12986_2022_708_MOESM1_ESM.docx]

**Figure S1.** A representative CT image at the third lumbar vertebra (L3) marked with different body composition. Skeletal muscle (SM) in red; subcutaneous adipose tissue (SAT) in blue; visceral adipose tissue (VAT) in yellow; intramuscular adipose tissue (IMAT) in green.


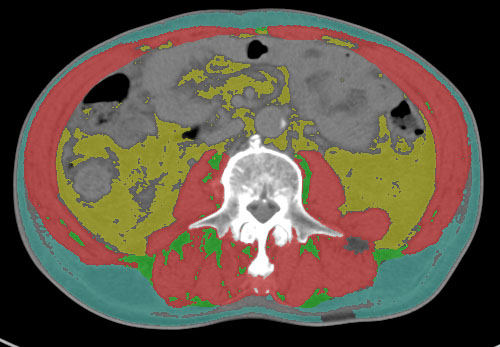


**Figure S2.** Gene expression distributions of each sample for RNA sequencing.


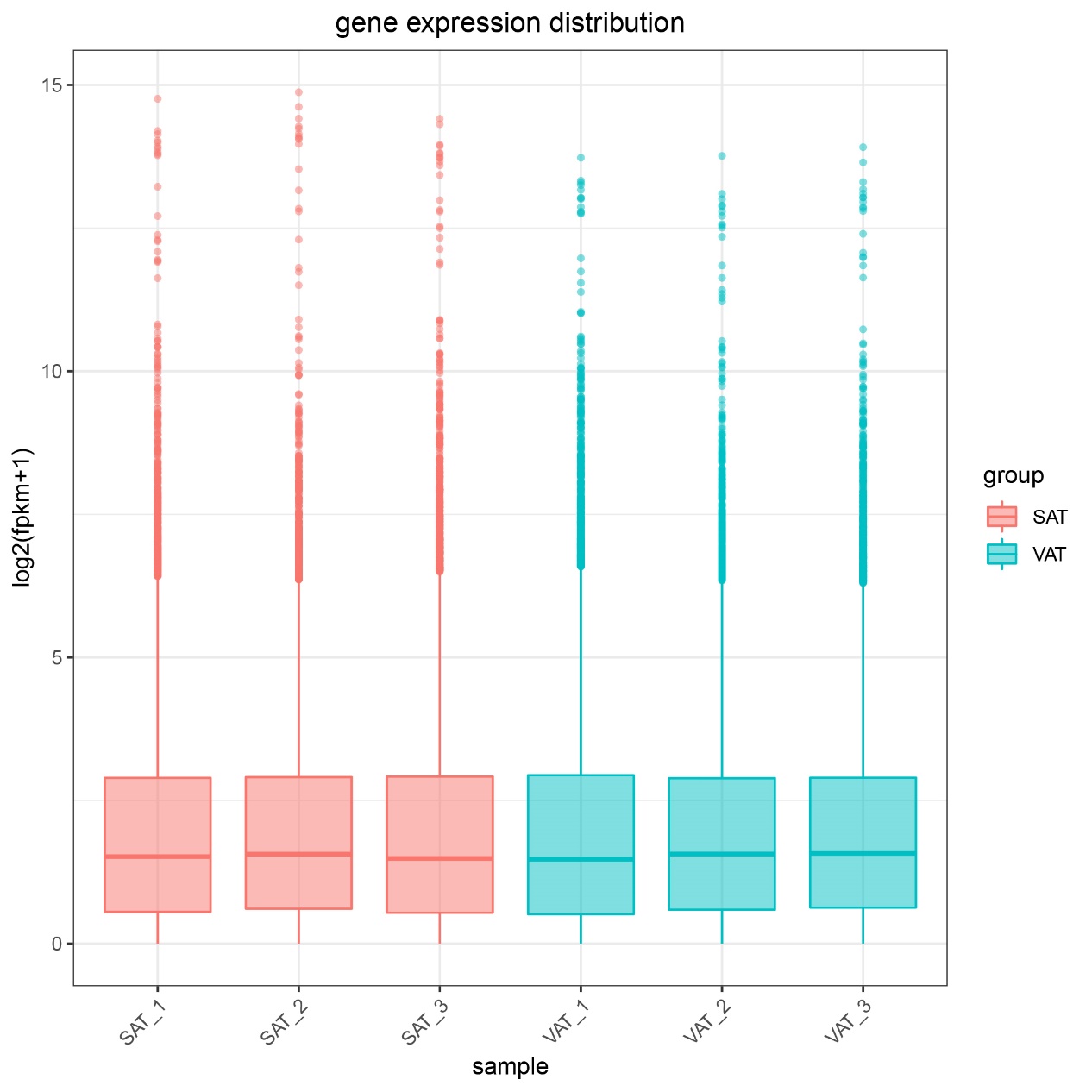


**Figure S3.** Representative images of SAT and VAT from normal and cachectic mice.


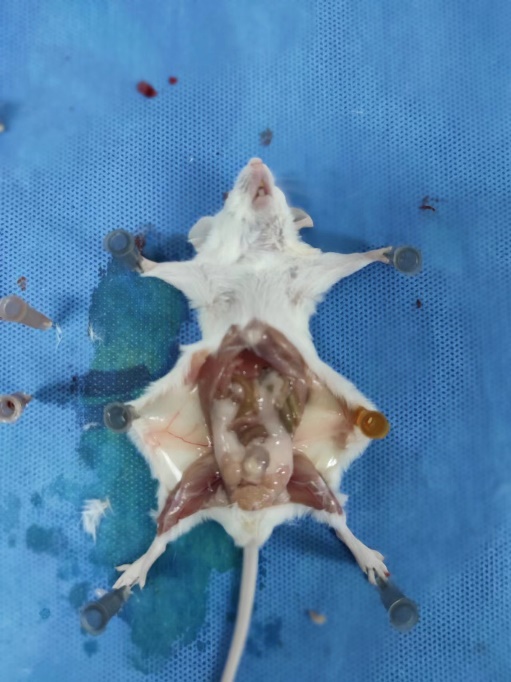

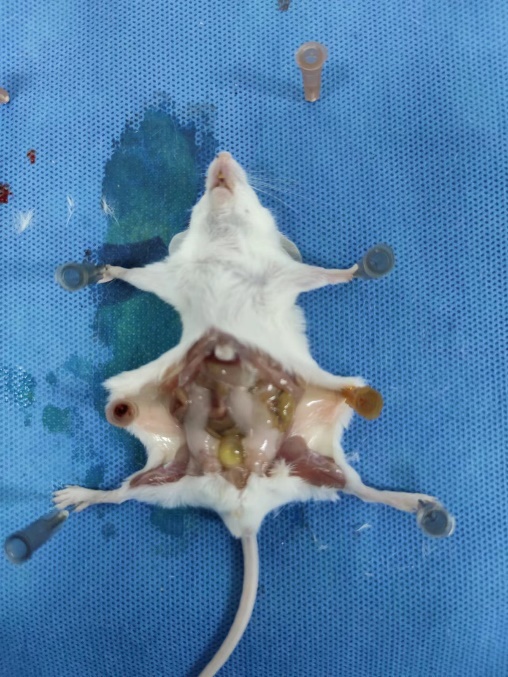


Cachexia

Normal
