# Supplementary material for: Analysis of different adipose depot gene expression in cachectic patients with gastric cancer
Source: Nutr Metab (Lond). 2022 Oct 31;19:72. doi: 10.1186/s12986-022-00708-x (PMC9624057; doi:10.1186/s12986-022-00708-x)
Supplement: Supplementary file 2 — Additional File 2. Table S1: Patients’ characteristics for RNA sequencing. Table S2: Primer Sequences used for qRT-PCR. Table S3: Clinical Characteristics of 61 cachectic patients. [file 12986_2022_708_MOESM2_ESM.docx]

**Table S1. Patients’ characteristics for RNA sequencing**

| Parameters | Cachexia (n = 3) |
| --- | --- |
| Ages (years) | 61.5±7.4 |
| Sex (n) | Male (3) |
| BMI (kg/m^2^) | 21.9±2.1 |
| Proportion of weight loss in recent 6 months (%) | 9.5±1.6 |
| Lymphocyte count (10^9/L) | 1.4±0.1 |
| Albumin (g/L) | 35.6±3.9 |
| IL-6 (pg/ml ) | 9.8±1.3 |
| TNF-α (pg/ml) | 11.2±1.8 |
| TNM stage III+IV(n) | 3 |
| Skeletal muscle area | 103.6±27.2 |
| VAT area (cm^2^) | 75.5±15.2 |
| SAT area (cm^2^) | 69.2±12.4 |

BMI, body mass index; IL-6, interleukin-6; TNF-α, tumor necrosis factor-α; SAT: subcutaneous adipose tissue; VAT: visceral adipose tissue

**Table S2. Primer Sequences used for qRT-PCR.**

| Name |  | Mouse |
| --- | --- | --- |
| ATGL | Forward | CAACGCCACTCACATCTACG |
|  | Reverse | AGCAGGCAGGGTCTTCAGT |
| HSL | Forward | CCTCAAAGTCAAACCCTCCA |
|  | Reverse | GTGCGTAAATCCATGCTGT |
| UCP1 | Forward | CACCTTCCCGCTGGACACT |
|  | Reverse | CCCTAGGACACCTTTATACCTAATGG |
| PRDM16 | Forward | ACTGAAGGAGGCCGACTTTG |
|  | Reverse | CGGAATGTGGGGTCCTCATC |
| CPT1 | Forward | TCGGTGAGCCTGGCCT |
|  | Reverse | TTGAGTGGTGACCGAGTCTG |
| AdipoQ | Forward | CCAATGTACCCATTCGCTTTAC |
|  | Reverse | GAAGTAGTAGAGTCCCGGAATG |
| FABP4 | Forward | CATCCGGTCAGAGAGTACTTTT |
|  | Reverse | TAGGGTTATGATGCTCTTCACC |
| CEBPα | Forward | AAGCCAAACAACGCAACGTG |
|  | Reverse | ACCAAGGAGCTCTCAGGCAG |
| GAPDH | Forward | TTGCCCTCAACGACCACTTT |
|  | Reverse | TGGTCCAGGGGTCTTACTCC |
| IRX1 | Forward | AGTATGAACTGAAGGACAACCC |
|  | Reverse: | ACCATAGGGATAATAAGCAGGC |
| β-ACTIN | Forward: | AGGTGTGCACCTTTTATTGGTCTCAA |
|  | Reverse: | TCCCTCTGGTTTGGAAGTATGT |

**Table S3. Clinical Characteristics of 61 cachectic patients**

| **Clinical**  **characteristics** | **Cachexia**  **(n=61)** |
| --- | --- |
| Gender(M/F) | 39/22 |
| Age | 65.13±8.74 |
| BMI | 20.93±2.05 |
| Weight Loss | 6.52±2.08 |
| IL6(mmol/L) | 9.44±5.52 |
| TNFa(mmol/L) | 13.28±5.67 |
| Alb (g/L) | 38.34±3.87 |
| PAb(mg/L) | 194.34±55.88 |
| FAA(mmol/L) | 0.56±0.15 |
| TC(mmol/L) | 4.10±0.99 |
| TG(mmol/L) | 1.09±0.39 |
| LDL(mmol/L) | 2.46±0.87 |
| HDL(mmol/L) | 1.21±0.45 |
| ApoA(g/L) | 1.16±0.27 |
| ApoB(g/L) | 0.81±0.23 |
| ApoE(mg/L) | 39.06±16.12 |
| SAT(cm^2^) | 115.84±21.02 |
| VAT(cm^2^) | 93.05±18.23 |

BMI Body mass index, ALB Albumin, PAb Prealbumin, TC Total cholesterol, TG Tri-glyceride, LDL Low-density lipoprotein, HDL High-density lipoprotein, ApoA Apolipoprotein A, ApoB Apolipoprotein B, ApoE Apolipoprotein E, FFA Free fatty acid, IL-6 Interleukin 6, TNF-α Tumor Necrosis Factor-α
